# A vision for cannabis regulation: a public health approach based on lessons learned from the regulation of alcohol and tobacco

**Published:** 2014-06-10

**Authors:** Mark Haden, Brian Emerson

**Affiliations:** Mark Haden, MSW, is Adjunct Professor at the University of British Columbia School of Population and Public Health and former Clinic Supervisor of the Raven Song Community Health Centre, Vancouver Coastal Health, Vancouver, BC.; Brian Emerson, MD, MHSC, is Chair of the Psychoactive Substances Committee, Health Officers Council of British Columbia, and Medical Consultant with the Population and Public Health Division, BC Ministry of Health, Victoria, BC.

There is growing evidence and awareness that the prohibition of cannabis is not achieving its purported objective of reducing use and potential harms, and instead has had considerable adverse consequences. 1–3 Uruguay, Colorado, and Washington State are jurisdictions where regulatory regimes not based in criminal law have recently been established for cannabis. However, there is widespread uncertainty regarding the potential benefits and harms of a non-prohibition–based regulatory framework for cannabis. This paper addresses this uncertainty by proposing a public health–oriented model for cannabis regulation that is derived from evidence-based recommendations for public health approaches to alcohol and tobacco control.

## Lessons learned from alcohol and tobacco control: a proposed regulatory model

A large body of research on alcohol- and tobacco- control measures to protect public health has been distilled in two key international evidence-based documents: *Alcohol: No Ordinary Commodity*, by Babor and colleagues^4^ and the WHO Framework Convention on Tobacco Control (FCTC).^5^ Drawing upon these sources, we constructed comparative tables organized according to the public health–oriented regulatory framework for psychoactive substances proposed by the Health Officers Council of British Columbia.^6^ This framework proposes controls with respect to availability, accessibility, supply, purchase, consumption, and use, as well as measures to reduce demand.

Tables 1 through 4 list evidence-based regulatory strategies for alcohol and tobacco from Babor and colleagues^4^ and the FCTC 5; these recommendations are also summarized in Box 1. In this article, we examine how these measures could be applied to cannabis. Where there are gaps in the regulatory recommendations, we propose measures that would be consistent with the objective of protecting public health.

**Table 1:** Availability and accessibility: evidence-based regulatory strategies for alcohol and tobacco

Policy category	Alcohol *	Tobacco†
Government monopoly on retail sales	Moderate effectiveness in limiting consumption and harm. Beneficial effects are increased by public health and public order goals.	Not mentioned.
Ban on sales	High degree of effectiveness in reducing consumption and harm, but often with adverse side-effects related to the black market, which is expensive to suppress. Ineffective without enforcement.	Not mentioned.
Hours and days of sale restrictions	Moderate effectiveness where changes in trading hours meaningfully reduce availability or where problems such as late-night violence are specifically related to hours of sale.	Not mentioned.
Restrictions on density of outlets	Moderate effectiveness for both consumption and social problems. Changes to outlet numbers affect availability most in areas with low prior availability, but bunching of outlets into high-density entertainment districts can be associated with public order problems and violence.	Not mentioned.
Sales by young people	Not mentioned.	Prohibit sales by people under a certain age. (Article 16, s. 7)
Taxes as a means to influence price	High degree of effectiveness in reducing consumption and harm. Effectiveness depends on government oversight and control of the total supply.	Implement tax and price policies that contribute to the health objectives aimed at reducing consumption, particularly by young people, and prohibit or restrict tax and duty-free importation by travellers. (Article 6, s. 1, 2)
Minimum price	No controlled studies / insufficient evidence. The logic of this strategy is based on price theory, but there is very little evidence of effectiveness. Competition regulations and trade policies may restrict implementation unless the minimum price is achieved through taxation policy.	Not mentioned.
Differential price by beverage	Limited effectiveness. Higher prices for distilled spirits shifts consumption to lower-alcohol content beverages, resulting in lower overall consumption. Evidence for the impact of tax breaks on lowalcohol products suggests a benefit.	Not mentioned.

* Effectiveness statements are based on Babor and colleagues, table 16.1, p. 240.^5^

† Paraphrased from the WHO Framework Convention on Tobacco Control.^6^

**Table 2:** Purchase, consumption, use: evidence-based regulatory strategies for alcohol and tobacco

Policy category	Alcohol *	Tobacco†
Legal purchase age	High degree of effectiveness in reducing traffic fatalities and other harms with minimal enforcement, but enforcement substantially increases effectiveness and cost.	Prohibit the sales of tobacco products to persons under a set age. These measures may include signage about the prohibition of tobacco sales to minors, requiring identification, banning direct access such as to store shelves, and ensuring that vending machines are not accessible to minors. (Article 16, s. 1)
Rationing	Moderate effectiveness, especially for heavy drinkers.	Not mentioned.
Size of purchase limitations	Not mentioned.	Prohibit sale of individual cigarettes or small packets that increase affordability for minors. (Article 16, s. 3)
Bans on public consumption	No controlled studies / insufficient evidence. Bans affect young or marginalized high-risk drinkers and may displace harm without necessarily reducing it.	Implement measures providing for protection from exposure to tobacco smoke in indoor workplaces, public transport, indoor public places and other public places. (Article 8, s. 1, 2)
Driving-related measures	Sobriety checkpoints: moderate effectiveness. Police campaigns are typically effective only in the short term. Deterrence is proportional to frequency of implementation and high visibility. Random breath tests: high degree of effectiveness. Effectiveness depends on the number of drivers directly affected and on the extent of consistent and high-profile enforcement. Lowered BAC limits: high degree of effectiveness. The lower the BAC limit, the more effective the policy. Very low BAC limits ("zero tolerance") are effective for youth and can be effective for adult drivers, but BAC limits below 0.02 are difficult to enforce. Administrative licence suspension: moderate effectiveness. When punishment is swift, effectiveness is increased. Effective in countries where it is applied consistently. Low BAC for young drivers: high degree of effectiveness. Clear evidence of effectiveness for those below the legal drinking or alcohol purchase age. Graduated licensing for novice drivers: moderate effectiveness. Can be used to incorporate lower BAC limits and licensing restrictions within one strategy. Some studies note that "zero tolerance" provisions are responsible for this effect. Severity of punishment: lack of effectiveness / limited effectiveness. Mixed evidence concerning mandatory or tougher sanctions for drunkdriving convictions. Effects decay over time in the absence of renewed enforcement or media publicity. Mandatory treatment of drunk-driving repeat offenders: limited effectiveness—punitive and coercive approaches have time-limited effects, and sometimes distract attention from more effective interventions.	Not mentioned.

BAC = blood alcohol concentration

* Effectiveness statements are based on Babor and colleagues, table 16.1, p. 240.^4^

† Paraphrased from the WHO Framework Convention on Tobacco Control.^5^

**Table 3:** Supply: evidence-based regulatory strategies for alcohol and tobacco

Policy category	Alcohol *	Tobacco†
Government control of production and manufacturing	Not mentioned.	Not mentioned.
Regulation of product constituents	Not mentioned.	Establish guidelines for testing and measuring contents and emissions, and for regulation of contents and emissions. (Article 9)
Regulation of product so it is not attractive to youth	Special or additional taxation on "alcopops" ("coolers" ) and other youth-oriented beverages: limited effectiveness—evidence that higher prices reduce consumption by young drinkers without complete substitution; no studies on impact on harms.	Prohibit manufacture and sale of sweets, snacks, toys or any other objects in the form of tobacco products that appeal to minors. (Article 16, s.1)

* Effectiveness statements are based on Babor and colleagues, table 16.1, p. 240.^4^

† Paraphrased from the WHO Framework Convention on Tobacco Control.^5^

**Table 4:** Demand: evidence-based regulatory strategies for alcohol and tobacco

Policy category	Alcohol *	Tobacco†
Restrictions on promotion (marketing, advertising, sponsorship, labelling, etc.)	Legal restrictions on exposures: limited/moderate effectiveness. There is strong evidence of a dose-response effect of exposure on young people's drinking, but evidence of only a small or insignificant effect on per-capita consumption from partial advertising bans; advertising bans or restrictions may shift marketing activities to less regulated media (e.g. Internet). Legal restrictions on content: no controlled studies / insufficient evidence. Evidence that advertising content affects consumption, but no evidence of the impact of content restrictions as embodied in industry self-regulation codes. Alcohol industry's voluntary self-regulation codes: lack of effectiveness. Industry voluntary self-regulation codes of practice are ineffective in limiting exposure of young persons to alcohol marketing, nor do they prevent objectionable content from being aired.	Not mentioned.Comprehensively ban advertising, promotion and sponsorship, including cross-border bans. If this is not possible, apply restrictions, including the prohibition of all forms of advertising, promotion, and sponsorship that promote a product by any means that is false, misleading, deceptive, or likely to create an erroneous impression about its characteristics, health effects, hazards, or emissions; require that warnings accompany all promotion; restrict the use of incentives that encourage purchase; require the disclosure of expenditures by the industry on promotion; restrict promotion on radio, television, print media the Internet; restrict sponsorship of international events. Ensure that product packaging and labelling do not promote a product by any means that are false, misleading, deceptive, or likely to create an erroneous impression about its characteristics, health effects, hazards or emissions, including by any means that directly or indirectly creates the false impression that one product is less harmful than others. These may include terms such as "low tar," "light," "ultra-light," or "mild". (Articles 11 and 13)
Bans on price discounts and promotions	No controlled studies / insufficient evidence: only weak studies in general populations of the effect of restrictions on consumption or harm; effectiveness appears to depend on availability of alternative forms of cheap alcohol.	Prohibit distribution of free products. (Article 16 s. 2)
Warning labels and signs	Lack of evidence of benefit. Labels and signs raise public awareness but do not change drinking behaviour.	Ensure that each package and any outside packaging and labelling carry health warnings describing the harmful effects and other appropriate messages. (Article 11 s. 1, 3, 4)
Information about product on packages	Not mentioned.	Each package and outside packaging and labelling shall contain information on relevant constituents and emissions. (Articles 10 and 11)

* Effectiveness statements are based on Babor and colleagues, table 16.1, p. 240.^4^

† Paraphrased from the WHO Framework Convention on Tobacco Control.^5^

Box 1 Summary of evidence-based regulatory strategies*

Availability and accessibility (see Table 1)

- Establish a government monopoly for retail sales.
- Place a ban on sales.
- Limit the hours and days of sales and restrict the number and density of commercial outlets.
- Prohibit sales to young people.
- Use pricing and taxation to influence consumption patterns.

Purchase, consumption, use (see Table 2)

- Establish a minimum purchase age.
- Limit maximum purchase quantities.
- Set minimum purchase quantities.
- Restrict smoking so that non-smokers are not affected.
- Implement impaired-driving measures.

Supply (see Table 3)

- Regulate product constituents and emissions.
- Ban modification of products to appeal to young people.

Demand (see Table 4)

- Prohibit or strictly limit product promotion.
- Include prominent health warning labels.
- Require disclosure of information about ingredients and emissions.

* From Babor and colleagues^4^ and the WHO Framework Convention on Tobacco Control 5

## Availability and accessibility

### Control structure

Experience has shown that a government monopoly can be effective in limiting alcohol consumption and related harms by (1) reducing the profit motive to promote sales and thereby encourage consumption; (2) reducing the political influence of special interests that would benefit from relaxed restrictions on availability; (3) limiting the number of sales outlets and their hours and days of business; and (4) having better-trained staff to reduce the likelihood of sales to minors.^7^ (See Table 1.)

We suggest that jurisdictions develop similar legislation and regulatory oversight with respect to cannabis, such as by establishing a governing body (e.g., a provincial "Cannabis Control Commission") with a clear mandate explicitly guided by public health goals. Generating government revenue should not be a primary driver of the policies of such a commission, which should operate at arm's-length from government to allow for stability and clarity of focus, to provide insulation from industry influence, and to resist the pressures of revenue-generation imperatives that would undermine the protection of public health.

The commission would control cannabis production, packaging, distribution, retailing, and revenue allocation and would play an important role in reducing demand. Processing and packaging would be done according to set standards in commission-licensed facilities. Direct sales from producers to retailers or consumers would not be allowed.

### Provision to consumers

Cannabis would be sold only through commission-operated or licensed outlets explicitly designed and required by law to support public health objectives. To minimize cannabis promotion, a standardized, neutral (i.e., bland-looking) and non-promoting environment for cannabis sales would be required. The clustering of cannabis outlets would not be allowed, as an aggregate presence could have undesirable effects on neighbourhoods, and outlets would be prohibited within 500 metres of a school, playground, or alcohol retail outlet.

Health promotion messages would be prominently displayed, and would include information about the laws against and risks of driving or operating heavy machinery while intoxicated. Information and referral mechanisms for cannabis dependency treatment would also be standardized and prominently displayed.

In line with evidence in relation to alcohol on the effectiveness of restricting the hours of sale (see Table 1), the hours of business of cannabis outlets would be limited.

### Price

There is strong evidence that taxation and price are important elements of a strategy to reduce alcohol consumption and tobacco use (see Table 1). Pricing and taxation policy should be balanced to establish a pricing structure that competes with the illegal market and allows for the needs of patients using cannabis for therapeutic purposes, while ensuring a sufficiently high price to restrict youth access and limit overall consumption.

## Purchase, consumption, use

### Purchase

A minimum purchase age for alcohol and tobacco products has been found to be an important strategy for controlling these substances (see Table 2). Similarly, the model for cannabis regulation that we propose would require sales to be limited to those over a specified age (e.g., 19). Purchases could involve filling out a form to access behind-the-counter cannabis; this could include a declaration that the cannabis is intended only for the purchaser or for others of legal age. Also, rationing has been found to be moderately effective, especially for heavy drinkers (see Table 2), and so we propose that customers would be allowed to make purchases only up to a certain amount (e.g., 10 grams a day). This small volume would also prevent the purchased cannabis from being diverted to young people or traded in an unregulated market.

### Cannabis use locations

The public use of alcohol and tobacco is contentious, and issues related to the public use of cannabis will no doubt arise in cannabis public use policy. Although public drinking is widely restricted in Canada, there is insufficient evidence of the public health effectiveness of bans on public drinking (see Table 2). With respect to tobacco, restrictions on the location of use are driven by the health hazards of environmental (second-hand) tobacco smoke. Given our lack of knowledge about the effects of environmental cannabis smoke—two recent reviews^2^,^8^ of health effects contain no mention of the specifc effects of cannabis smoke—and the public health concern about exposure to any type of smoke, we propose that cannabis smoking be restricted to licensed locations or to private homes. The health of workers at cannabis use locations could be protected by providing separate, ventilated spaces for customers and prohibiting cannabis smoking by workers on shift.

Cannabis lounges should have a standardized, neutral, external and internal appearance, should be free of promotional materials or activities, and should display health promotion and referral information prominently. These locations would thus also offer the opportunity for public health promotion by providing a central, accessible, and social venue through which information dissemination and demonstration of potential harm reduction and health promotion approaches can occur, such as encouraging the use of smokeless modes of cannabis consumption that may reduce exposure to particulates.^9^

To support the public health objective of separating cannabis, alcohol, and tobacco consumption, no alcohol or tobacco use should be permitted in public cannabis use locations.

Consumption locations would obtain their supply from the commission, would be permitted to sell to customers, would have restrictions on the size of the outlet and its days and hours of operation, and would be required to establish "good neighbour" agreements. Training would be required in recognizing and intervening with people experiencing problems related to their consumption patterns. No "special price reductions" or "happy hour discounts" would be permitted.

## Supply

Although Babor and colleagues^4^ and the FCTC^5^ provide no guidance with regard to public health–oriented regulatory recommendations for the supply of alcohol and tobacco, supply management is an implicit feature of the government monopoly favoured for public health purposes and has been strongly recommended as a component of a public health approach to tobacco.^10^,^11^

### Production

To control supply, the commission would be the only organization authorized to purchase cannabis from licensed growers, to import it into a province, and to supply retailers. Supply management systems similar to agriculture marketing boards could be established to manage the supply and protect small producers. People would be allowed to grow cannabis for their own personal consumption but not to resell it; this would be similar to the home brewing of beer and wine, which does not require a licence. To legally grow cannabis for the purpose of selling it would require a licence and adherence to processes to ensure quality and safety. This model of for-profit private growers with controlled distribution and retailing is similar to the provincial or state alcohol monopolies and models that have been proposed for tobacco.^10^,^11^

Many public health problems are determined by social and economic factors,^12^ particularly unequal wealth distribution.^13^ An equitable approach to the distribution of cannabis-related wealth that supported many small-scale growers and producers and prevented large concentrations of wealth by multinational corporations would be consistent with the promotion of public health goals: the formulation of cannabis policy should be alert to the potential for multinational corporations to economically exploit the legitimization of the cannabis trade and subsequently exert profit-motive-driven pressure on public health policy related to cannabis control.

### Product

The FCTC requires that constituents and emissions of tobacco products be regulated (see Table 3). Similar requirements should be applied to cannabis. The concentration of the psychoactive ingredient delta- 9-tetrahydrocannabinol (THC) has been noted to have increased over the years,^14^ likely for a variety of reasons (e.g., increased effect per dose, easier storage and transport). This parallels the availability of concentrated alcohol products that emerged during the Prohibition era, when illegal dealers preferred to import and transport spirits rather than beer and wine because moving smaller volumes helped them avoid detection.^15^ Concentrated products increase the risk of harm and are often not preferred by users. It has been observed in the Netherlands, where cannabis is de facto legal, that users prefer relatively lower THC concentrations. 16 In this model, retailers could sell a variety of strains with clearly labelled concentrations of THC in both smokable and edible products.

Only bulk products should be made available, to allow individuals to determine their dose rather than being exposed to a predetermined per-unit dose, as is the case with manufactured cigarettes. This would also prevent the potential for attractively marketing cannabis as cigarette-like products. Processed products (e.g., tinctures, cookies) packaged in child-proof containers and prepared according to specific regulatory requirements should also be available to avoid the harms of smoke inhalation.

## Demand drivers

### Promotion and packaging

Recommendations to limit advertising, promotion, and sponsorship as a means of reducing psychoactive substance use and harms are well supported by research evidence (see Table 4). This suggests that one of the most important lessons of the commercialization of tobacco and alcohol is that product promotion is a significant driver of consumption and related harms. Branding of products is critical to promotion—and, once branding is allowed, promotion is very difficult to prevent. Therefore, all branding and promotion of cannabis products should be prohibited, and plain packaging should be required (i.e., no logos, brand names, or colourful packaging).

Labelling about product constituents and health risks are considered important to prevent the harms of tobacco (see Table 4). For cannabis, the packaging should describe the concentration of important constituents and the strain, and should include dominant, standardized warning labels that mention the respiratory irritation of inhaling smoke, using cannabis with alcohol, using cannabis while driving or operating other machinery.

### Public education

Demand could be tempered through evidence-based public and school education, but such efforts should avoid large public anti-cannabis prevention campaigns, which have been shown to have the potential to unintentionally stimulate interest in and actually increase the use of cannabis.^17^,^18^

## Dedicated revenue

The revenue raised from cannabis regulation should be used for health and social initiatives such as early childhood development, education, housing for marginalized people and improving mental health and addictions services.

## Conclusion

Public support for cannabis "legalization" is growing, in part because of increasing recognition of the lack of effectiveness and the harms of cannabis prohibition, together with the pressing need for proactive measures based on a public health approach. Otherwise, a commercial exploitation model may result, such that public health and social problems similar to those associated with alcohol and tobacco will be repeated.

In Canada there are legal mechanisms that could allow a cannabis regulation pilot project in a province without violating federal laws, such as by obtaining a Controlled Drugs and Substances Act^19^ section 56 exemption (see Box 2) and/or using the exemption and regulation provisions of section 55. Such exemptions could allow a province to establish a province-level scientific project, explicitly guided by public health oriented goals and objectives, with allowance for specific demonstration sites in accepting communities.

Changes to cannabis regulation will require detailed analysis grounded in the experience with alcohol and tobacco as described by Rolles,^20^ and must include rigorous evaluation to monitor for unintended consequences, potential harms, and anticipated benefits of a new regime.

Box 2 ***Controlled Drugs and Substances Act,* section 56^19^**

The Minister may, on such terms and conditions as the Minister deems necessary, exempt any person or class of persons or any controlled substance or precursor or any class thereof from the application of all or any of the provisions of this Act or the regulations if, in the opinion of the Minister, the exemption is necessary for a medical or scientific purpose or is otherwise in the public interest.
